# Heredity in Disease

**Published:** 1900-04-21

**Authors:** 


### The Hospital

A Journal of

The Medical Sciences and Hospital Administration.

Vol. XXVIII.—No. 708.] Edited by Sir Henry Burdett, K.C.B., and Solomon C. Smith, M.D., M.R.C.P. [April 21, 1900.

### Heredity in Disease.

If, in trying to explain the origin of our diseases, we often put the blame upon our ancestors, we are well supported in so doing by those high authorities who call heredity to their aid in almost every biological difficulty. And, indeed, heredity must be held responsible for much. There can be no doubt that, just as we derived our lives from our parents, and they from theirs, so from our ancestors do we derive our tendencies, our stature, the conformation of our heads, the structure and activity of our livers, the mode in which we digest and assimilate our food, and our tendency to run through our span of life in few or many years. All these we derive not perhaps from, but rather through our ancestry, and according as that ancestry has been "select" or mongrel, so shall we, or shall we not, possess strongly-marked family peculiarities as to feature and strongly-marked family proclivities in relation to disease. Modern life, no doubt, tends to the production of mongrels. The abolition of local limitation in the selection of mates, the mixture of race with race which is encouraged by modern means of locomotion, the general topsy-turveydom which makes it so easy to sink from social heights, or rise from social mud —all these tend not exactly to a levelling, but rather to an admixture of types which in another hundred years or so will probably wipe out many of those hereditary endowments which have made men of one district to have long hands, and men in another round ones, and have perpetuated family peculiarities through a long series of generations. "Breed" does not entirely depend on fathers. We all have fathers. It rather depends on a long series of forefathers, surrounded by the same influences; and if, as seems not impossible, the mongrel should in future predominate, this will arise quite as much from lack of permanence in social position as from intermixture of social strata. Perhaps in the future disease-tendencies, like man himself, will also be of mixed or mongrel type, interlarded here and there with strong instances of reversions cropping up according to the law of atavism. Let us then hasten to study heredity in disease while "types" still remain amongst us.

Professor Hamilton, in opening a discussion on heredity in disease at the Edinburgh Medico-Chirurgical Society, pointed out that it must be regarded as an hereditary tendency to break down under certain influences, a vulnerability of certain tissues, transmitted in the same way as any other hereditary peculiarities. If, then, we are to accept current hypothesis as to the continuity of the germ-plasm according to which the germ-cells are the exclusive bearers of hereditary qualities, and the perishable somatic cells have no influence in that direction, except so far as they may produce inequalities in the nutrition and thus in the structure of the germ-plasm, we see that it is no more possible for a man, by excess or otherwise, to originate a disease such as gout, or "set up" a tendency, such as an instability of the nervous system, which shall be transmitted to his descendants, than it is possible by the exercise of his muscles or of his virtues to endow future generations with either the one or the other. The question of heredity in disease is on all fours with the much-debated problem, "Can acquired characteristics be transmitted?" to which the Weissmannists all say "No."

Taking as an example the question of tuberculosis, no doubt some part of the well-recognised family tendency to this disease is due to the fact that in a tuberculous family the chances of infection are greater than among other surroundings, but "every physician and surgeon knows," says Professor Hamilton, "that the members of such families are, in a large number of instances, notable as tuberculous subjects from their conformation of bodies." "My argument," he says, "is that it is this habit of bod[illegible] not the disease itself, which is inherited. . . . Granted, for the sake of argument, that this habit of body which is handed on from one generation to another is the cause of the hereditary disposition to tuberculosis . . . where has the inherited strain come from? . . . Can it be generated by vicious surroundings? I question whether it can. . . . My own impression is that these features are the lineal descendants of a variation which took place far back in our history, that the variation has occurred irrespective of surroundings or external agencies, and that its influence has been propagated in the descendants ever since." The same with gout, and the same again with a host of nerve derangements which are hereditary enough in one sense, but not in the sense that the sins of the individual fall upon his children, as the saying is, to the third and fourth generation. The position taken among biologists at the present day would seem, then, to be that the various hereditary tendencies or predispositions to disease of hereditary type have arisen, like other transmitted peculiarities, as variations in the germ-plasm, and that these predispositions probably extend far back into the human race. This is consoling enough to those who, little as they may care for *convenances*, are upset at the thought that their descendants may suffer from their acts; but it is the opposite of consoling to those who hope, by striving to leave the world a little better than they found it, to make a permanent improvement in the human race. Vain hopes and idle fantasies! The future springs out of the far past, and we are bound in unending fetters by the "continuity of germ-plasm."

